# Traditional Food, Health, and Diet Quality in Syilx Okanagan Adults in British Columbia, Canada

**DOI:** 10.3390/nu12040927

**Published:** 2020-03-27

**Authors:** Rosanne Blanchet, Noreen Willows, Suzanne Johnson, Okanagan Nation Salmon Reintroduction Initiatives, Malek Batal

**Affiliations:** 1 Department of Agricultural, Food & Nutritional Science, Faculty of Agricultural, Life & Environmental Sciences, University of Alberta, Edmonton, AB T6G 2P5, Canada; 2 Okanagan Nation Alliance, West Kelowna, BC V4T 3L7, Canada; 3 Department of Nutrition, Faculty of Medicine, University of Montreal, P.O. Box 6128, succ. Centre-ville, Montreal, QC H3C 3J7, Canada; malek.batal@umontreal.ca; 4 Centre de recherche en santé publique du Québec (CReSP), Montreal, QC H3C 3J7, Canada

**Keywords:** First Nations, traditional food, ultra-processed food, ultra-processed products, diet, nutrient intake, nutrition transition, Indigenous food sovereignty

## Abstract

In Canada, store-bought food constitutes the majority of First Nations (FN) people’s diets; however, their traditional foods (TF; wild fish, game, fowl, and plants) remains vital for their health. This study compares health indicators and diet quality among 265 Syilx Okanagan adults according to whether or not they reported eating TF during a 24-h dietary recall. Three methods assessed diet quality: nutrient intakes and adequacy, Healthy Eating Index (HEI-C), and contributions of ultra-processed products (UPP) to %energy using the NOVA classification. Fifty-nine participants (22%) reported eating TF during the dietary recall; TF contributed to 13% of their energy intake. There were no significant differences in weight status or prevalence of chronic disease between TF eaters and non-eaters. TF eaters had significantly higher intakes of protein; omega-3 fatty acids; dietary fibre; copper; magnesium; manganese; phosphorus; potassium; zinc; niacin; riboflavin; and vitamins B6, B12, D, and E than non-eaters. TF eaters also had significantly better diet quality based on the HEI-C and the %energy from UPP. Findings support that TF are critical contributors to the diet quality of FN individuals. Strength-based FN-led interventions, such as Indigenous food sovereignty initiatives, should be promoted to improve access to TF and to foster TF consumption.

## 1. Introduction

Around the world, including in Canada, Indigenous peoples suffer from health disparities. First Nations, Metis, and Inuit, the three groups of Indigenous peoples recognized by the Government of Canada [1], face a higher prevalence of obesity, cardiovascular disease, diabetes, mental health issues, and arthritis, a lower life expectancy, and overall poor health outcomes compared with non-Indigenous people living in Canada [2]. These inequalities are rooted in unfavourable structural determinants of health [2].

Traditional foods (TF) of First Nations peoples include fresh or minimally processed foods obtained from their local environments, such as wild fish, game, fowl, roots, berries, and other plants [3]. Before contact with European settlers and during the early-colonial periods, TF were the only food source of First Nations peoples [3]. Sustainable TF harvesting was predicated on First Nations people’s prudent ecosystem management of their territories. In this way, food harvesting provided reliable sustenance, food security, and food sovereignty and connected First Nations (FN) peoples to their traditional ways of knowing and being [4,5]. Nowadays, market foods, defined as foods that are purchased at the store, constitute the majority of First Nations people’s diets [4,6]. Although nutritious market foods could complement TF, food and drinks of poor nutritional quality contribute to a large portion of First Nations people’s contemporary diets [6,7]. This nutrition transition to a “Western diet” is a consequence of multiple colonial policies that forcibly led to diminished traditional food harvesting and more sedentary lifestyles [4,5]. This dietary shift has resulted in excessive intakes of energy, carbohydrates, fat, and sodium and inadequate intakes of several nutrients (e.g., dietary fibre; calcium; iron; folate; and vitamins A, D, and E), which, coupled with physical inactivity, has led to a high prevalence of obesity, diabetes, cardiovascular disease, and overall ill health [3,6,7,8,9,10,11].

Despite significant nutrition, lifestyle, and environmental changes, TF remain vital for many First Nations peoples, because they contribute important nutrients to the diet even when consumed in small amounts [3,8,12,13,14]. In addition, eating TF continues to be important for First Nations people’s spiritual, cultural, social, psychological, and economic well-being [3,4]. The harvesting of TF continues to play a significant role in promoting physical activity, strengthening a sense of cultural identity, and connecting people to their traditional territories [8]. Considering the required recognition of First Nations people’s jurisdiction and responsibility over the environmental stewardship and well-being of the land and those who depend on it to harvest TF, nutrition research that continues to document the important function of TF for FN peoples is needed [15].

In nutrition research, comprehensive methods to assess diet quality have been developed recently in addition to studying the intake of single nutrients to reflect diet complexity and diversity of the nutritional quality of market foods (e.g., fresh potato versus potato chips) [6,13]. For instance, the Healthy Eating Index (HEI-C) assesses the quality of the diet using Canadian nutrition guidelines [16], and the NOVA classification categorizes foods by the degree of food processing in order to determine the contribution of ultra-processed products (UPP) to the total daily dietary energy intake (%E (%energy) from UPP) [17]. Both indices have been used to evaluate the impact of diet quality on health-related outcomes in Canada [13,18]. However, only a few studies have used diet quality scores such as the HEI-C and the %E from UPP to assess the diets of Indigenous peoples in Canada [6,13]. There is a need to analyze the diet of First Nations peoples with more specificity to better understand the impact of market foods and traditional foods on health and diet quality [8].

Historically, Syilx Okanagan (referred to as Syilx from now on) individuals were subsistence fishers, hunters, and gatherers [19]. Their diet was mostly composed of wild salmon, game, berries, and plants [19,20]; however, colonization, the dispossession of Indigenous land, and oppressive policies and practices, along with urbanization and habitat degradation, resulted in the extirpation of many traditional food species and Syilx poor health outcomes [20]. The diet of Syilx adults has never been assessed using a single nutrient assessment or comprehensive diet quality measures. The objectives of the present study were: (1) to describe present TF consumption in a First Nation in Canada, namely among Syilx adults; (2) assess the association of TF consumption with health indicators; and (3) compare diet quality of TF eaters and non-eaters using three methods: nutrient intakes and nutrient adequacy, the HEI-C, and the %E from UPP using the NOVA classification. This research is important for the Syilx Okanagan Nation to advocate for enhanced access to their TF. Understanding the relationship between TF intakes, health, and nutrition of Syilx adults might lead to interventions to increase TF in the diet of Syilx people, such as food sovereignty and food security initiatives.

## 2. Materials and Methods

### 2.1. Study Sampling and Data Collection

This study is a component of the Okanagan Salmon and Our Health Study, which is a joint project between the Okanagan Nation Alliance, University of Alberta, Université de Montréal, and Syilx communities. The objective of the overarching study is to document the health and health equity outcomes of the Syilx Okanagan Nation initiatives that led to the reintroduction of Okanagan sockeye salmon (*Oncorhynchus nerka)* in the Okanagan River upstream of Osoyoos Lake. The Syilx Okanagan Nation transverses the 49th parallel, which is the international border between Canada and the United States. The study occurred solely in Canadian communities, specifically in the Okanagan region of British Columbia, Canada. The Syilx Okanagan Nation in Canada comprises seven communities.

First Nations Principles of Ownership, Control, Access and Possession (OCAP^®^) [21], as well as the Canadian “Tri-Council Policy Statement: Ethical Conduct for Research Involving Humans” [22], guided the study. Participating communities, the Okanagan Nation Alliance, University of Alberta, and Université de Montréal signed community research agreements that ensured respectful and equitable power-sharing relations between parties. Study activities were planned with the involvement of participating communities and the Okanagan Nation Alliance. Participating communities and the Nation own their data and are involved in the interpretation of research findings. Data, results, and reports were discussed with community members to ensure their cultural acceptability before being widely distributed. All participants provided written informed consent. This study was conducted according to the guidelines laid down in the Declaration of Helsinki. The Research Ethics Office of the University of Alberta (Pro00067679) and the Research Ethics Committee of the Université de Montréal (16-074-CERES-D) granted ethics approval for the study.

All Syilx communities were invited to participate in the study; three agreed to do so. Data collection was conducted between February and August 2018 in these three Syilx communities. Within each of them, households were either all selected (communities with <250 households) or randomly selected (communities with ≥250 households). Interviewers were instructed to select the person with the next birthday when there was more than one eligible adult in a household. Criteria for inclusion were: 19 years of age and older, self-identified as Syilx or in a kin relationship with a person who self-identified as Syilx, and living in a Syilx community or in a town located next to a Syilx community. Exclusion criteria included being pregnant or breastfeeding.

Community members were hired as interviewers in each community. Interviewers participated in a 2-day training session offered by the project coordinator, who is a registered dietitian with previous experience conducting nutrition research with Indigenous communities. Training covered sampling frame, eligibility criteria, the survey, and how to conduct 24-h dietary recalls. Interviewers administered the questionnaire to collect information on dietary patterns, lifestyle, health status indicators, and food security using handheld Android tablets that automated data collection. Dietary intakes were collected manually using a 24-h dietary recall [23] and reviewed by the project coordinator. Participants received a CAD $50 gift certificate to acknowledge their contribution, time, and cost associated with participation. Participants received CAD $25 for second dietary recalls.

### 2.2. Health Indicators

Participants were invited to have their weight and height measured. Those who agreed were measured, lightly dressed and without shoes, using the World Health Organization (WHO) guidelines [24]. Height was measured with a measuring tape to the nearest millimetre (mm). Body weight was collected with a calibrated digital scale (Seca 803, Hamburg, Germany) to the nearest 0.1 kg. Measurements were done twice; the mean value of the two measurements was calculated. Body mass index (BMI) was calculated as weight/height^2^ (kg/m^2^), and weight status was classified according to WHO BMI categories [24]. Participants who did not agree to have their anthropometric measurements taken were invited to report them. There was no statistically significant difference between measured and reported BMI; therefore, data for measured and reported BMI were combined for the present study. Participants were asked if they had ever been told by a healthcare provider that they had hypertension, diabetes, or cardiovascular disease (yes/no), and they reported on their overall health status (poor, fair, good, very good, or excellent). These health indicators were selected given the high prevalence of chronic disease among FN in Canada [2] and so that the present study could report comparable data as the Canada-wide First Nation Food, Nutrition and Environment Study [25].

### 2.3. Dietary Assessment

Dietary intake data were collected using a 24-h dietary recall, employing a 3-stage multiple pass method: quick list, detailed description, and review with estimation of portion sizes [23], with the aid of 3-dimensional food models (Santé Québec, Montréal, QC, Canada) and household measures. Information on cooking methods and brand names were obtained whenever possible. A second dietary recall was completed for 19% of the participants to determine usual nutrient intakes and nutrient adequacy. Nutritional supplements were included in the dietary recalls.

A nutrition student in her fourth year of university studies and a registered dietitian entered foods from the 24-h dietary recalls into ESHA Food Processor SQL version 11.0.137 (ESHA Research, Salem, OR, USA). Dietary recalls were analyzed using nutrient contents from the Canadian Nutrient File [26] and other sources (e.g., the US Department of Agriculture database). Some new products were manually added using the information provided by the manufacturers. The accuracy of dietary recalls was ensured using two steps: (1) a review of all records by the project coordinator who is a registered dietitian and (2) a review of outliers such as unusual foods and intakes that were ±2 standard deviations (SD) of the means for energy and selected nutrients.

Software of Intake Distribution Estimation (SIDE-IML; Iowa State University, Ames, IA, USA) was used to determine the distribution of usual nutrient intakes from foods, beverages, and nutritional supplements; these were compared to age- and sex-specific dietary reference intakes (DRIs). Macronutrient intakes were compared with the acceptable macronutrient distribution ranges (AMDR), which are expressed as a percentage of total energy intakes [27]. Other nutrients were compared with the estimated average requirement (EAR), when one was available, or adequate intake (AI), using the cut-point method [28]. Iron intakes of women aged 19–50 years old were assessed using the probability approach [28]. Sodium intakes were compared to the chronic disease risk reduction threshold [29].

The HEI-C is an adaptation of the American Healthy Eating Index, where the diet is compared to ten recommendations assessing two aspects of diet quality: adequacy and moderation [16]. The HEI-C components reflect major recommendations from 2007 Eating Well with Canada’s Food Guide [16,30]. The HEI-C has not been updated to represent the 2019 Canada’s Food Guide, which has similar healthy eating recommendations. The HEI-C scores vary from 0 to 100, with higher scores representing better diet quality [16].

The NOVA classification categorizes foods and drinks into four groups according to the nature and extent of food processing: Group 1, unprocessed or minimally processed (e.g., fresh fruits, vegetables, roots, legumes, meat, fish, unsalted nuts, eggs, etc.); Group 2, culinary ingredients (e.g., oils, sugar, salt, etc.); Group 3, processed products (e.g., canned vegetables, fruits, or legumes; salted, sugared, canned, or cured meat and fish; and sweetened or salted nuts, etc.); and Group 4, ultra-processed products (UPP; e.g., carbonated drinks, salty or sweet snacks, commercial breads, cereals or energy bars, etc.) [17]. All foods and drinks reported in the 24-h dietary recalls were classified into these four groups, and mean estimates of the proportion of energy (%E) were calculated from each food group. Higher %E coming from UPP are indicative of a lower diet quality [6].

The first dietary recall was used to classify participants as TF eaters if at least one TF was reported to have been eaten. Usual nutrient intakes and nutrient adequacy (based on the first recalls and the subset of individuals with two recalls), the HEI-C and the %E from UPP (both calculated from the first recalls) were used to assess diet quality.

### 2.4. Statistical Analyses

Statistical analyses were conducted using SAS 9.4 (SAS Institute Inc. Cary, NC, USA). Geometrical means and log-normal transformations were performed for variables that were not normally distributed. Frequencies (%) and means were used to describe the socioeconomic status of participants. Chi-square tests and *t*-test analyses were performed to assess differences in demographic characteristics and health status indicators between adults who did and did not consume TF during the 24-h recall. Two sets of logistic regression analyses were conducted to test the association between TF consumption and health indicators: one solely adjusted for age and the other adjusted for age, gender, and clustering of participants in communities. Differences in nutrient intakes, HEI-C, and %E from UPP between TF eaters and non-eaters were examined using analysis of covariance (ANCOVA) with Bonferroni adjustment to take into account multiple tests. Adjustments were made for the following variables: age, sex, and clustering of participants in communities. Analyses on micronutrient intakes and HEI-C were further adjusted for energy intakes. Proportions of participants who met the dietary reference intakes (DRIs) were compared between TF eaters and non-eaters with chi-square tests. The HEI-C scores and the %E from UPP were also classified into quartiles, because they are derived from a single 24-h dietary recall, which is a good measure of group intakes but not individual intakes [31]. The first quartile of HEI-C included the lowest HEI-C scores (lowest diet quality), and the fourth quartile included the highest HEI-C scores (highest diet quality). The first quartile of the %E from UPP included those who ate UPP the least (highest diet quality), and the fourth quartile included those who consumed UPP the most (lowest diet quality). Quartiles of HEI-C and %E from UPP were compared between TF eaters and non-eaters using chi-square tests. A *p*-value < 0.05 was considered to be statistically significant.

## 3. Results

Of the 561 households that were selected to participate, 329 were contacted (58.6%) by community lay interviewers. Among households that were contacted, three households were not eligible, and six homes were vacant. Of the 320 eligible households, 265 adults completed the interview, for a participation rate of 82.8%.

Mean age of participants was 49.8 (standard error (SE) 1.0) years, and 70.2% were women (Table 1). Fifty-nine participants (22.2%) reported having eaten at least one TF during the 24-h reference period. TF eaters were significantly older than non-eaters (*p* = 0.0205). There were no significant differences in gender proportion or health indicators (i.e., BMI, weight status, nutrition-related chronic diseases, or self-reported health status) between TF eaters and non-eaters. Similarly, none of the associations between TF and any health outcomes were significant in adjusted logistic regression models (data not shown).

A total of 21 different TF items were reported by 59 participants during the 24-h dietary recall (Table 2). Four TF were eaten by >15% of participants: sockeye salmon (*Oncorhynchus nerka,* 32.2%), deer (*Odocoileus hemionus or Odocoileus virginianus*; 27.1%), moose (*Alces alces*, 15.3%), and Saskatoon berries (*Amelanchier alnifolia*, 15.3%).

Mean energy intake of participants was 2094.0 kcal/day (SE 34.4; Table 3). TF contributed an average of 12.9% (SE 1.8%) of the energy intake of participants who ate TF. TF eaters had significantly higher intakes of protein (%E); omega-3 fatty acids (%E); dietary fibre; copper; magnesium; manganese; phosphorus; potassium; zinc; niacin; riboflavin; and vitamins B6, B12, D, and E (Table 3 and Table 4). TF eaters were also more likely to meet the estimated average requirement for copper and vitamins C and D (Table 5).

Scores from the HEI-C ranged from 20.3 to 92.7, with a mean of 50.5 (SE 0.8) out of a maximum score of 100 (Table 6). Participants who reported eating TF during the 24-h recall had higher HEI-C scores (mean 53.9, SE 1.8, range 27.5–86.9), indicating a better diet quality, than their counterparts who did not eat TF (mean 47.4, SE 1.2, range 20.3–92.7). TF eaters also had a significantly higher likelihood of having a HEI-C score in the higher quartile, representing a superior diet quality (*p* = 0.0257).

**Figure 1:**
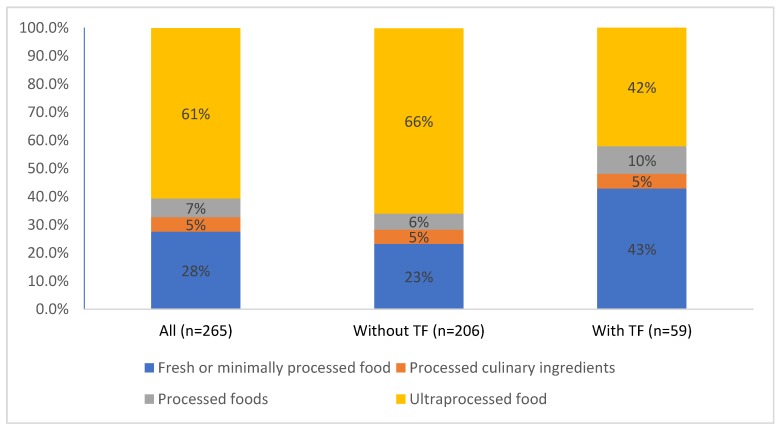
Proportion of food energy from the 4 NOVA food categories according to whether or not participants reported eating traditional foods (TF) using 24-h recalls.

Figure 1 shows that, on average, UPP accounted for 60.6% of energy (SE 1.5%, range 0–100%), unprocessed or minimally processed foods for 27.6% of energy (SE 1.3%, range 0–87.5%), processed products for 6.6% of energy (SE 0.6%, range 0–62.0%), and culinary ingredients for 5.1% of energy (SE 0.5%, range 0–64.1%). Participants who ate TF had lower %E from UPP (mean 42.6%, SE 3.0, range 0–87.3%) than participants who did not eat TF (mean 66.0%, SE 2.0%, range 0–100%), an indicator of better diet quality (Table 6). They also had higher %E from fresh and minimally processed foods (TF eaters: 52.4% and TF non-eaters: 17.0%). Participants who ate TF were similarly more likely to be in the quartile with the lowest %E from UPP (*p* < 0.0001).

## 4. Discussion

When European settlers arrived in what is now British Columbia, Canada, the Syilx were a thriving Nation, as attested to by the size of their traditional territory [19]. The literature [32] and oral history support that their diet was adequate to maintain good health. A nutrition transition to a Western diet was largely a forced act of survival for the Syilx Okanagan Nation, as colonial policies of land and traditional foods dispossession reduced the accessibility of traditional foods while contributing to ecosystem changes that affected livelihood and food availability [19]. In the present study, almost one-quarter of Syilx adults ate TF during the 24 h preceding their survey, indicating the cultural significance of these foods. Although TF contributed to a low proportion of the daily energy intake of Syilx adults, similar to previous studies with other Indigenous populations in Canada, TF consumption was significantly associated with enhanced nutrient intakes [3,7,10,25,33,34,35,36,37,38], higher HEI-C scores [39], lower %E from UPP [6,8,39], and higher %E from fresh and minimally processed foods [6,8,39]. Findings from the current study therefore corroborate that TF consumption, even in small quantities, is associated with a better diet quality in Indigenous peoples in Canada [3,7,12,14,33,40,41]. Willows et al. (2018) suggested that TF eaters have a healthier diet, because TF are more nutrient dense than market foods and because TF replace UPP in the diet [8], which is supported by the current study. It is possible that participants who ate TF may have been more likely to make home-prepared meals and less likely to purchase UPP. Based on the better diet quality of TF eaters, it could be hypothesized that they would have a lower prevalence of obesity and diet-related chronic disease than TF non-eaters. However, this was not observed in the present study, possibly because energy intakes were not lower among TF eaters, TF eaters consumed excess quantities of UPP like their TF non-eating counterparts, and/or TF consumption was assessed solely on one day, not taking the quantity of food eaten into account.

Significantly higher intakes of protein (%E); omega-3 fatty acids (%E); dietary fibre; copper; magnesium; manganese; phosphorus; potassium; zinc; niacin; riboflavin; and vitamins B6, B12, D, and E were observed among participants who had eaten TF during the reference period. In addition, TF eaters were more likely to meet the dietary reference intakes for copper, vitamin C, and vitamin D. Although some differences in nutrient intakes were small in absolute terms, these differences could have a long-term positive impact on the nutritional health of Syilx TF eaters [33,39]. Of note, vitamin D and omega-3 fatty acids intakes were substantially higher among TF eaters, which could be explained by salmon consumption, the most eaten traditional Syilx food item and an important source of these two nutrients.

When the quality of the diet was classified using HEI-C, the average score of Syilx participants was 50.5, which is on the threshold between a diet of poor quality and a diet that requires improvement but is not of poor quality. This score is almost identical to the score obtained among First Nations adults who participated in the national First Nations Food Nutrition and Environment Study (FNFNES) in Canada [39]. When Syilx participants in the current study were categorized based on TF consumption, participants who did not eat TF had a HEI-C score of 47.4 qualifying their diet as of poor quality, whereas the score of 53.9 for TF eaters implied a diet that required improvement but was not of poor quality.

Evidence from Canada has shown that UPP should contribute less than one-third of the energy intake to reach nutrient goals for the prevention of obesity and nutrition-related chronic disease [18]. The contribution of UPP to energy (60.6%) in the present study is well above that recommendation. This proportion is also higher than what was observed in *Eeyou Istchee* First Nation adults in the Province of Quebec [8,13] and in First Nation adults who participated in the FNFNES [6]. Higher UPP intake could be explained by the very close proximity of participating Syilx communities to urban centres (FN communities were in remoter locations in the other studies. However, Syilx participants who ate TF had lower %E from UPP (42.6%) than reported in the other studies [6,8,13]. Considering that UPP are related to poor nutrition and health [13,18,42,43,44,45], lower contributions of UPP to daily energy intake is likely going to positively impact the health and well-being of TF eaters.

The proportion of TF eaters and the contribution of TF to their total daily energy intake were similar to what has been observed among First Nations adults who participated in the FNFNES [6]. Similar to previous studies [7,8,41,46,47], older people were more likely to have eaten TF than younger ones. In the present study, there were no differences in the proportion of TF eaters between men and women, which is similar to findings among Dene and Metis in the Northwest Territories [7] and among Innu in Quebec [47] but different than in *Eeyou Istchee* adults (Cree First Nation in Quebec, Canada) [8,41]. The different findings related to gender could be explained by the season in which the surveys were conducted and by the types of TF consumed. Women may have poorer access to meat and fowl than men (hunting is still mostly done by men), which would be the main TF items eaten during winter, whereas women may have better access to plants and berries during the spring/summer harvesting season, which is when the current study was conducted. It is also possible that the Okanagan Nation Alliance contributes to gender equity in TF access with its food fishery and community fish distribution program implemented as a result of the restoration of the salmon habitat [19,20]. Recently, numerous ecosystem rehabilitation initiatives by the Nation have resulted in a renewed Okanagan sockeye salmon (*Oncorhynchus nerka*) fishery [19]. That sockeye salmon was the most commonly eaten TF item among study participants is a testimony to the efforts of the Nation to bring back this culturally significant food species, also potentially resulting in better nutrition and diet quality for Nation members. The Nation also supports hunting camps and mentors Syilx Okanagan members to go on the land. The findings of the present study indicate that First Nations’ environmental initiatives to steward their lands, waters, and air will likely result in positive nutritional outcomes for community members.

### Limitations

When interpreting the present findings, one should take the study limitations into account. The three communities that agreed to participate in the study may be different from the four other Syilx communities in Canada. As this study was community-engaged research, local community members were hired to administer surveys to increase community capacity and because community members were knowledgeable of Syilx foods, cultural practices, and the language (Nsyilxcen). Although community interviewers were instructed to contact all households assigned to them, only 58% of selected households were contacted for unknown reasons. The omissions may have introduced selection bias, which could limit generalizability of the findings to all Syilx adults. Furthermore, fewer men than women participated in the present study, and therefore, the results may not be representative of the general male Syilx population [48]. About half of anthropometric measurements and all other health indicators were self-reported, which may have resulted in under- or overestimation of their prevalence. Nutritional supplements were included in nutrient intakes, which may have influenced total nutrient intakes and/or the difference in nutrient intakes between TF eaters and non-eaters. However, this would not have affected findings related to HEI-C scores or the %E from UPP. Social desirability bias (i.e., valuing fresh foods more than processed foods) may have resulted in the underestimation of unhealthy foods in the diet and the overestimation of healthy foods such as TF that are promoted in Syilx communities [28]. One 24-h recall is a good measure of group intakes but cannot be used to characterize usual intakes in individuals [28,31]. For this reason, we used usual intakes for nutrients and categorized the HEI-C and proportion of energy intake from UPP in quartiles. There is some possibility of error and missing information on nutrients in the nutrient databases. It is possible that some foods were misclassified into the NOVA food classification due to limited food choices in the nutrient files because the classification was applied a posteriori. However, this bias was reduced, because the project coordinator took the NOVA classification into account as much as possible when she revised data entry and changed entries to better reflect the processing of the foods eaten. It is also possible that ingredients in mixed foods entered without recipes, such as margarine used in making a traditional First Nations’ quick bread called bannock, may have been included in the fresh and minimally processed foods [6]. The HEI-C does not discriminate foods based on their processing level, which is a limit of this index. Lastly, the group of TF consumers was relatively small, which may affect the robustness of the statistical analyses. Log-normal transformations of data that were not normally distributed helped to ensure robustness of the statistical analyses [49].

## 5. Conclusions

As shown in the current study, the nutritious Syilx traditional diet has nowadays been replaced by mostly UPP among Syilx adults, with negative consequences for their nutrition. Yet, importantly, our study found that consuming even a low amount of traditional foods was associated with better diet quality using three different assessment methods. These results support the vital importance of traditional foods for the diet quality of Syilx adults and reinforce the requirement to increase access to TF in Indigenous communities of Canada. Further research should use strength-based approaches to support interventions enabling traditional food intakes among Indigenous peoples, such as Indigenous food sovereignty initiatives.

## Figures and Tables

**Table 1:** Characteristics of participants according to whether or not they ate at least one traditional food (TF) using 24-h recalls.

Characteristics	All Participants (*n* = 265)	No TF in Diet (*n* = 206)	TF in Diet (*n* = 59)	*p*
Gender (%)				0.5186
Women	70.2	66.1	71.4	
Men	29.8	33.9	28.7	
Age (years), mean ± SE	49.8 ± 1.0	48.6 ± 1.1	54.1 ± 2.0	0.0205
BMI (kg/m^2^), mean ± SE*	31.0 ± 0.5	30.9 ± 0.5	31.5 ± 1.0	0.5494
Weight status (%)				0.6851
Normal-weight	17.6	14.6	18.5	
Overweight	27.3	25.0	28.0	
Obesity	55.1	60.4	53.6	
Diabetes (%yes)	14.8	13.6	19.0	0.3006
Hypertension (%yes)	21.5	21.6	21.1	1.0000
Cardiovascular disease (%yes)	5.1	6.0	1.8	0.3085
Health status				0.2307
Poor/Fair	29.7	32.2	21.1	
Good	38.2	36.1	45.6	
Very good/excellent	32.1	31.7	33.3	

SE: standard error; *Measured or reported, *n* = 216.

**Table 2:** Traditional food items reported eaten at least once on 24-h recalls by 59 Syilx adults who ate traditional food.

Species	*n*	Proportion of Participants (%)
Sockeye salmon	19	32.2
Deer	16	27.1
Moose	9	15.3
Saskatoon berries	9	15.3
Blueberries	7	11.9
Asparagus	4	6.8
Strawberries	4	6.8
Huckleberries	3	5.1
Gooseberries	2	3.4
Bison	1	1.7
Blackberries	1	1.7
Black currants	1	1.7
Elk	1	1.7
Camas	1	1.7
Chanterelle mushrooms	1	1.7
Stinging nettle leaves	1	1.7
Raspberries	1	1.7
Pink Salmon	1	1.7
Maple syrup	1	1.7
Labrador tea	1	1.7
Rainbow trout	1	1.7

**Table 3:** Energy and macronutrient intakes^a^ as a percentage of total energy intakes of participants, according to whether or not they reported eating traditional food (TF) using 24-h recalls.

Variables	All Participants^b^ (*n* = 265)	No TF in Diet^c^ (*n* = 206)	TF in Diet^c^ (*n* = 59)	*p*
Energy (kcal)	2094.0 ± 34.4	2134.0 ± 47.3	2159.4 ± 72.8	0.7460
Protein (%E)	15.9 ± 0.1	15.5 ± 0.2	16.8 ± 0.3	<0.0001
Carbohydrates (%E)	49.3 ± 0.4	49.7 ± 0.6	48.9 ± 1.0	0.4332
Sugar (%E)	20.2 ± 0.4	20.5 ± 0.6	19.6 ± 0.9	0.2985
Fat, total (%E)	34.6 ± 0.5	34.4 ± 0.6	33.4 ± 0.9	0.2729
SFA (%E)	11.2 ± 0.1	11.2 ± 0.2	10.6 ± 0.3	0.0779
MUFA (%E)	11.8 ± 0.1	11.6 ± 0.2	11.7 ± 0.3	0.8432
PUFA (%E)	7.2 ± 0.1	7.1 ± 0.2	7.1 ± 0.3	0.8750
Omega-3 fatty acids (%E)	0.8 ± 0.0	0.8 ± 0.0	0.9 ± 0.0	0.0003
Omega-6 fatty acids (%E)	6.0 ± 0.1	6.0 ± 0.2	5.8 ± 0.3	0.4443

kcal: kilocalories; %E: percentage of daily energy intake; SFA: saturated fatty acids; MUFA: monounsaturated fatty acids; PUFA: polyunsaturated fatty acids; ^a^ nutrient intakes include intakes from foods, beverages, and nutritional supplements; ^b^ values are arithmetic or geometric means ± standard errors; and ^c^ values are least square means ± standard errors adjusted for the following variables: age, sex, and clustering of participants in communities.

**Table 4:** Fibre, cholesterol, and micronutrient intakes^a^ of participants according to whether or not they reported eating traditional foods (TF) using the 24-h recall.

Variables	All Participants^b^ (*n* = 265)	No TF in Diet^c^ (*n* = 206)	TF in Diet^c^ (*n* = 59)	*p*
Fibre (g)	17.5 ± 0.4	17.0 ± 0.5	18.7 ± 0.7	0.0237
Calcium (mg)	685.0 ± 12.6	667.7 ± 15.2	676.1 ± 23.4	0.7379
Copper (mg)	1.1 ± 0.0	1.1 ± 0.0	1.2 ± 0.0	0.0003
Iron (mg)	13.8 ± 0.2	13.6 ± 0.2	14.1 ± 0.4	0.2720
Magnesium (mg)	273.0 ± 4.2	267.0 ± 4.6	290.8 ± 7.1	0.0020
Manganese (mg)	3.2 ± 1.0	3.1 ± 1.0	3.7 ± 1.0	0.0042
Phosphorus (mg)	1128.0 ± 23.3	1094.7 ± 22.5	1193.6 ± 34.6	0.0085
Potassium (mg)	2511.0 ± 48.6	2380.5 ± 51.2	2629.7 ± 78.7	0.0036
Sodium (mg)	3235.0 ± 63.9	3265.5 ± 66.0	3092.4 ± 101.6	0.1151
Zinc (mg)	9.8 ± 9.8	9.6 ± 0.3	11.0 ± 0.4	0.0013
Vitamin A (μg RAE)	442.0 ± 1.0	411.0 ± 1.0	445.7 ± 1.0	0.1679
Folate (μg DFE)	378.0 ± 6.6	375.1 ± 8.0	376.4 ± 12.4	0.9241
Niacin (mg NE)	33.7 ± 1.0	33.0 ± 1.0	36.0 ± 1.0	0.0035
Pantothenic acid (mg)	5.8 ± 1.0	5.9 ± 1.0	6.2 ± 1.0	0.2263
Thiamin (mg)	1.5 ± 1.0	1.5 ± 1.0	1.5 ± 1.0	0.8789
Riboflavin (mg)	2.0 ± 1.0	2.0 ± 1.0	2.4 ± 1.0	0.0006
Vitamin B6 (mg)	1.7 ± 1.0	1.6 ± 1.0	1.8 ± 1.0	0.0280
Vitamin B12 (μg)	4.1 ± 1.0	3.8 ± 1.0	5.3 ± 1.1	<0.0001
Vitamin C (mg)	77.8 ± 1.0	73.6 ± 1.0	84.9 ± 1.1	0.0930
Vitamin D (mg)	3.0 ± 0.0	2.9 ± 1.1	3.7 ± 1.1	0.0259
Vitamin E (μg)	7.7 ± 1.0	7.4 ± 1.0	8.5 ± 1.0	0.0234
Cholesterol (mg)	328.0 ± 6.1	258.5 ± 7.8	253.4 ± 12.0	0.07397

g: grams; mg: milligrams; ug: micrograms; RAE: retinol activity equivalent; DFE: dietary folate equivalent; NE: niacin equivalent; ^a^ nutrient intakes include intakes from foods, beverages, and nutritional supplements; ^b^ values are arithmetic or geometric means ± standard errors; and ^c^ values are least square means ± standard errors adjusted for the following variables: age, sex, energy intake, and clustering of participants in communities.

**Table 5:** Proportion of traditional food (TF) eaters and non-eaters meeting the dietary recommended intakes (DRIs)*.

Nutrient	DRI	% Meeting DRI All* (*n* = 265)	% Meeting DRI No TF in Diet* (*n* = 206)	% Meeting DRI TF in Diet* (*n* = 59)	*p*
Protein	AMDR	100	100	100	NA
Carbohydrates	AI	99.6	100	98.3	0.0612
Carbohydrates	AMDR	63.5	75.2	78.0	0.6665
Fat, total	AMDR	50.5	49.0	61.0	0.1042
Fiber	AI	10.6	9.2	15.3	0.1840
Calcium	EAR	22.6	22.8	22.0	0.8893
Copper	EAR	94.5	91.8	100	0.0226
Iron	EAR	95.9	94.2	96.7	0.2270
Magnesium	EAR	38.9	36.4	47.5	0.1247
Manganese	EAR	88.7	86.9	94.9	0.0864
Phosphorus	EAR	95.5	94.7	100	0.0698
Potassium	AI	32.5	31.1	37.3	0.6383
Sodium	CDRR	17.1	18.0	18.6	0.9044
Zinc	EAR	72.5	70.4	79.7	0.1598
Vitamin A	EAR	37.0	36.9	37.0	0.9558
Folate	EAR	70.5	68.0	67.8	0.9810
Niacin	EAR	100	100	100	NA
Pantothenic acid	AI	69.5	62.0	76.3	0.1045
Thiamin	EAR	87.6	87.9	86.4	0.7703
Riboflavin	EAR	97.0	96.6	98.3	0.5002
Vitamin B6	EAR	81.5	80.6	84.8	0.4677
Vitamin B12	EAR	87.1	85.44	94.9	0.0519
Vitamin C	EAR	61.5	57.3	76.3	0.0082
Vitamin D	EAR	5.7	3.4	13.6	0.0029
Vitamin E	EAR	18.2	15.3	20.3	0.3819

AI: adequate intake; AMDR: acceptable macronutrient distribution ranges; CDRR: chronic disease risk reduction; EAR: estimated average requirement; NA: nonapplicable; and *proportion of participants within AMDR ranges, >EAR, >AI, <CDRR.

**Table 6:** Diet quality indices of participants according to whether or not they reported eating traditional foods (TF) during the 24-h recall.

Variables	All Participants (*n* = 265)	No TF in Diet (*n* = 206)	TF in Diet (*n* = 59)	*p*
Healthy Eating Index (HEI-C)				
Score	50.5 ± 0.8^a^	47.4 ± 1.2^b^	53.9 ± 1.8^b^	0.0010
Quartiles				0.0257
Quartile 1 (lowest scores)	25.3	28.2	15.3	
Quartile 2	24.5	26.2	18.6	
Quartile 3	25.3	24.3	28.9	
Quartile 4 (highest scores)	24.9	21.4	37.3	
Proportion of energy (%E) from UPP				
Score	60.6 ± 1.5^a^	66.0 ± 2.0^c^	42.6 ± 3.0^c^	<0.0001
Quartiles				<0.0001
Quartile 1 (lowest %E from UPP)	25.3	17.0	54.2	
Quartile 2	24.9	24.8	25.4	
Quartile 3	24.9	28.2	13.6	
Quartile 4 (highest %E from UPP)	24.9	30.1	6.8	

UPP: ultra-processed products; ^a^ values are arithmetic or geometric means ± standard errors; ^b^ values are least square means ± standard errors adjusted for the following variables: age, sex, energy intake, and clustering of participants in communities; ^c^ and values are least square means ± standard errors adjusted for the following variables: age, sex, and clustering of participants in communities.
